# Enhanced Photocatalytic Properties of Restacked Unilamellar [SrTa_2_O_7_]^2–^ Nanosheets of Aurivillius Phase Layered Perovskites

**DOI:** 10.1021/acsomega.3c00593

**Published:** 2023-03-09

**Authors:** Mohammadreza Khodabakhsh, Bengisu Yilmaz, Sadegh Firoozi, Davoud Fatmehsari Haghshenas, Ugur Unal

**Affiliations:** † Chemistry Department, Koc University, Rumelifeneri yolu, Sariyer, 34450 Istanbul, Turkey; ‡ Department of Materials and Metallurgical Engineering, Amirkabir University of Technology, No. 350, Hafez Ave, Valiasr Square, 1591634311 Tehran, Iran; § Koc University Surface Science and Technology Center (KUYTAM), Koc University, Rumelifeneri yolu, Sariyer, 34450 Istanbul, Turkey; ∥ Koc University Tupras Energy Center (KUTEM), Koc University, Rumelifeneri yolu, Sariyer, 34450 Istanbul, Turkey; ⊥ Materials Science and Engineering Department, Koc University, Rumelifeneri yolu, Sariyer, 34450 Istanbul, Turkey

## Abstract

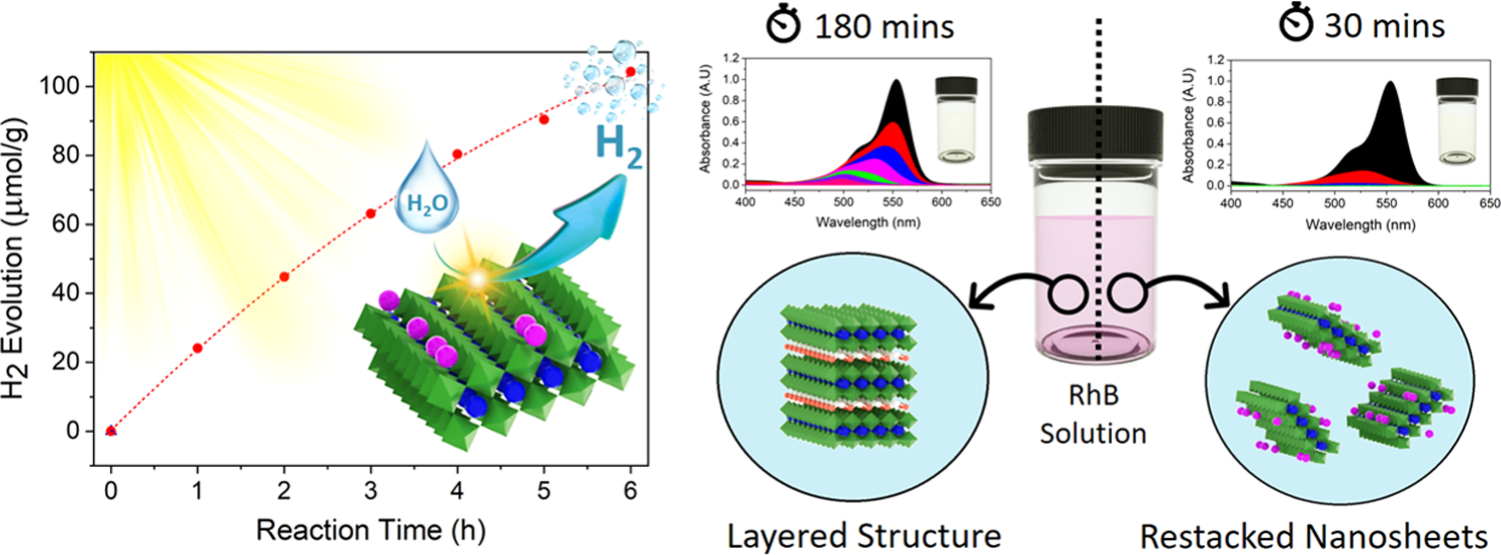

In the present work, unilamellar [SrTa_2_O_7_]^2–^ perovskite nanosheets with variable lateral dimensions were synthesized via a high-yield, three-step liquid exfoliation route from layered Bi_2_SrTa_2_O_9_. The photocatalytic activity of the parent and exfoliated layered perovskites was evaluated for the photocatalytic dye degradation of Rhodamine B under UV light (254 nm) and reduction of water to H_2_ under the full solar spectrum. A comparative study of the photocatalytic behavior of unilamellar [SrTa_2_O_7_]^2–^ perovskite nanosheets and parent layered structure showed a significant improvement in both hydrogen evolution (98.20 vs 3 μmol g^–1^) and Rhodamine B degradation time (180 vs 30 min), with the restacked nanosheets. The exfoliation of layered perovskites not only increases their specific surface area, providing more active sites, but also reduces the recombination probability of electrons and holes due to their unilamellar structure and reduced charge transport pathways. The synthesis and preparation of strong acid solids such as [SrTa_2_O_7_]^2–^ perovskite nanosheets can be a promising approach for effective adsorption of pollutants with cationic nature and more efficient electron transfer between the dye and catalyst. Finally, the photocatalytic characteristics of the restacked unilamellar [SrTa_2_O_7_]^2–^ nanosheets remained unchanged after three successive cycles of recycling–reusing.

## 1 Introduction

Shortly after the first report on using oxide semiconductor photocatalysts by Fujishima and Honda,^1^ many successful studies have been conducted to improve the efficiency of photocatalytic reactions, with a particular focus on the development of visible light-responsive photocatalysts through band gap modification.^2−4^ In addition to conventional approaches for changing the band gap of semiconductors, researchers have focused on the design and synthesis of alternative structures to reveal the relationship between the morphology and optical properties of the nanomaterials.^5−7^ It is worthy of note that photocatalysis is a surface reaction in which both electrons and holes have to reach the surface of the structure without recombination and/or trapping on the defect sites.^8^ The chance of electron–hole recombination decreases by reducing the particle size regarding the shorter travel distance to reach the surface.^9,10^ Reportedly, increasing the active sites by manipulating the morphology, more specifically the structures with a high aspect ratio and large surface area, has a significant effect on the photocatalytic activity of the catalyst.^11−16^ Bismuth layered perovskites (Bi_2_A_*n*–1_B*_n_*O_3*n*+3_), also known as Aurivillius perovskites, are one of the three members of the layered perovskite family. Their general formula can also be written in the form of [Bi_2_O_2_]^2+^[A_*n*–1_B*_n_*O_3*n*+1_]^2–^, as the perovskite blocks are separated by [Bi_2_O_2_]^2+^ sheets with a fluorite structure. These layered perovskites have recently attracted much attention due to their unique structure that can prevent the electron–hole recombination depending on the congregation of electrons and holes on different surfaces.^17,18^ Just like the two other members of the layered perovskite family (i.e., Ruddleson–Popper and Dion–Jacobson), Aurivillius perovskites have a distinctive advantage over other types of oxides, which is the possibility to be exfoliated into nanosheets.^19,20^ However, a high-yield exfoliation process is still a challenging issue.^21−23^ In this research, not only we attempt to develop an efficient exfoliation procedure for Aurivillius perovskites but also the photocatalytic activity of the obtained restacked [SrTa_2_O_7_]^2–^ nanosheets is compared with that of the initial parent layered structure (Bi_2_SrTa_2_O_9_) in photocatalytic hydrogen evolution and degradation of Rhodamine B dye. Bi_2_SrTa_2_O_9_ as a member of the Aurivillius family consists of two-dimensional (2D) negatively charged [SrTa_2_O_7_]^2–^ perovskite blocks interleaved with [Bi_2_O_2_]^2+^ sheets. For the exfoliation of stacked perovskite slabs into single nanosheets, selective acid leaching (protonation) of interlayer cations and subsequent intercalation with bulky alkali molecules, i.e., alkyl ammonium molecules, are two necessary steps.^19,24−26^ The “exfoliation–restacking” technique has become a convenient method to increase the photocatalytic reaction sites using the interlayer space of layered materials.^27−29^ This approach can significantly increase the specific surface area of the photocatalyst material, and it will shorten the charge transport pathways, reducing the chance for electron–hole recombination. This enhances the photocatalytic performance of our material and reduces the reaction time.^30,31^ In addition, another advantage of the perovskite nanosheets over the layered structure is their negative surface charge, which provides the electrostatic attraction toward the cationic dyes such as Rhodamine B; in other words, this attractive interaction facilitates the dye adsorption by the perovskite nanosheets.^32^

A survey of the previous literature on photocatalytic characteristics of Aurivillius perovskites indicates that the morphology/structure of the reported Aurivillius perovskites is multilayered or sheet-like; more specifically, the Bi_2_O_2_ interlayers still remained between the perovskite layers; in technical parlance, they are not exfoliated and cannot be considered single perovskite 2D nanosheets.^16,33^ In comparison to the previous reports on Aurivillius phase perovskites, our three-step intercalation process not only produces unilamellar nanosheets but also yields larger nanosheets in size (50 nm–1 μm).^21,23,34−36^ The main objective of this work was to develop an efficient procedure for the exfoliation of Aurivillius perovskites and to evaluate the photocatalytic activity of the restacked [SrTa_2_O_7_]^2–^ nanosheets for the hydrogen evolution reaction and degradation of Rhodamine B dye. Additionally, the study aimed to determine the extent of improvement in photocatalytic activity resulting from the structural manipulation by examination of the original layered structure (Bi_2_SrTa_2_O_9_) activity under the studied photocatalytic conditions.

## 2 Experimental Section

### 2.1 Layered Structure and Nanosheet Preparation

**Figure 1:**
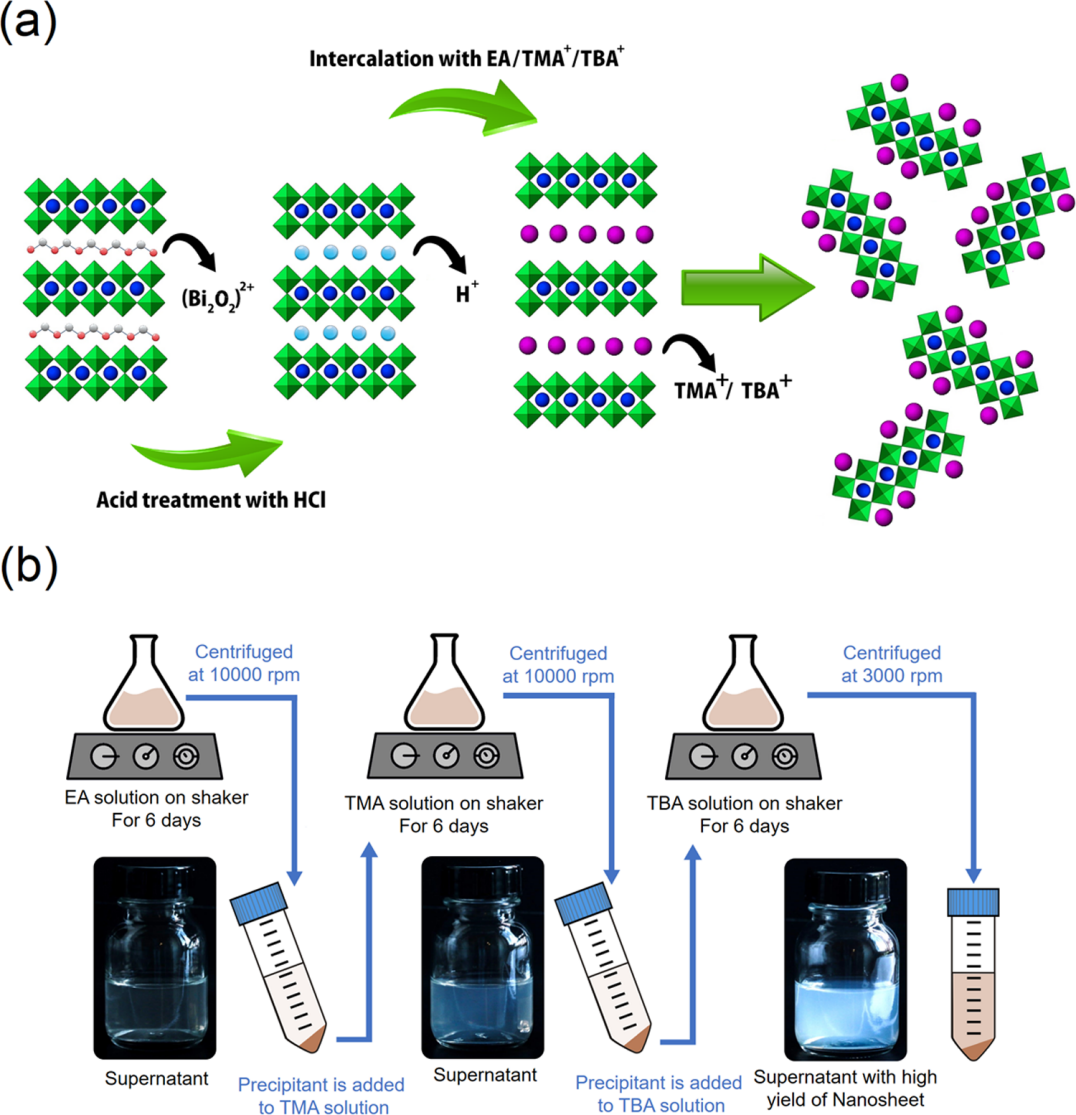
Mechanism of protonation, intercalation, and exfoliation of the double-layered Aurivillius perovskites (a) and schematic of the three-step exfoliation procedures (b).

The double-layered bismuth strontium tantalite (Bi_2_SrTa_2_O_9_) powder (BST) was prepared through a solid-state reaction of bismuth oxide (Bi_2_O_3_ Alfa Aesar 99.99%), strontium carbonate (SrCO_3_ Alfa Aesar 99.99%), and tantalum oxide (Ta_2_O_5_ Alfa Aesar 99.9%) as the precursors. A 500 mg mixture of the above-mentioned precursors, with the stoichiometric ratio, was mixed and milled with 40 g of zirconia balls (diameter of 0.5 cm) for 12 h. For uniform distribution of the precursors in the mixture, ethanol (15 mL) was introduced into the milling system. Afterward, the ball-milled mixture was transferred to an alumina crucible and heated through a three-step calcination procedure at 900, 1050, and 1200 °C, with a heating rate of 3 °C/min for 4 h. Between each step of calcination, the mixture was ground to achieve higher uniformity and to prevent the formation of undesired phases. A proton exchange reaction was performed by stirring the parent structure in 6 M HCl solution for 7 days under ambient conditions using an orbital shaker, and the acid solution was refreshed every 2 days to ensure complete leaching of [Bi_2_O_2_]^2+^ layers. Then, the protonated powder was separated centrifugally (8000 RPM/8600 RCF) and washed with distilled water until the pH reached ∼7. Finally, the obtained powder was dried under ambient conditions. As depicted in Figure 1a, to exfoliate the protonated powder, three-step exfoliation protocol was carried out in 50 mL of ethylamine solution (EA, Merck 70% aqueous solution), 50 mL of tetramethylammonium hydroxide solution (TMA^+^OH^–^, Merck 25% in water), and 50 mL of tetrabutylammonium hydroxide solution (TBA^+^OH^–^, Alfa Aesar 40% aqueous solution) for 6 days each. It should be pointed out that after the first step of exfoliation (i.e., washing with EA for 6 days), the powder was separated centrifugally (10 000 RPM/13 400 RCF for 30 min) and it was added to TMA without drying. The same separation and collection procedure was employed after the second step, and the obtained precipitant was introduced into the TBA, and finally after 6 days, the nanosheet colloids and nonexfoliated particles were separated by centrifugation (3000 RPM/1200 RCF for 30 min). Many studies have reported restacking exfoliated nanosheets by changing the ionic strength or pH of the colloidal solution. For this purpose, various acids such as HCl are used since the negatively charged nanosheets can electrostatically combine with protons to reconstruct the protonated form of the layered structure.^28,37−40^ To restack exfoliated nanosheets, a few drops of diluted HCl solution (0.1 M) were added to the nanosheet colloid and then, the colloid was centrifuged at 5000 RPM/3350 RCF and dried at 80 °C. To remove TMA^+^/TBA^+^ cations from the surface of nanosheets, the final precipitate was washed few times with distilled water until the pH reaches ∼7 and then dried under ambient conditions. The absence of TMA or TBA cations was confirmed by FTIR measurements (Figure S1).

### 2.2 Film Preparation

For the photoelectrochemical experiments, all three forms of the prepared material (the as-synthesized powder, protonated powder, and restacked nanosheets) were coated on the fluorine-doped tin oxide (FTO)-coated glass. Prior to the deposition, the FTO substrates were ultrasonically cleaned with acetone and ethanol for 10 min each with intermediate water rinsing. After drying the substrates under a N_2_ flow, the FTO substrates were coated (by the as-synthesized powder and protonated powder) via electrophoretic deposition based on a layer-by-layer (LBL) technique, whereas a single layer of 2D nanosheets was coated on a FTO substrate by the dip coating method.

For electrophoretic deposition, 10 mg of the prepared powders and 5 mg of iodine (Sigma-Aldrich, 99.8% ACS reagent) were ultrasonically mixed into 30 mL of acetone in a beaker. The FTO-coated glasses were used both as the cathode and an anode. The distance between the cathode and anode was ∼1 cm, and the deposition was conducted at 30 V for 3 min using a DC power supply (RIGOL DP811). After coating, the films were rinsed with deionized water to remove excess iodine and then dried under a N_2_ flow. For the dip coating method, the cleaned FTO substrates were pretreated in an aqueous solution of PEI (2 g/L) for 15 min. After rinsing with deionized water, the PEI-coated FTO substrates were dipped into the nanosheet colloid (0.5 mg/mL) for 15 min and then rinsed with deionized water.

### 2.3 Structural Characterization

The crystalline structure of the powders was studied with X-ray diffraction (XRD) using a Bruker/D8 Advance with Cu Kα radiation. The elemental analysis of the synthesized and restacked nanosheets was carried out using a Bruker Tiger S8 X-ray fluorescence spectrometer (XRF). The atomic force microscopy (AFM) images and height profile of the exfoliated unilamellar nanosheets were collected using a Bruker Dimension Icon instrument and high-sensitivity silicone Bruker RTESPA MPP-11120-10 probes with a nominal spring constant of 40 N/m. The recorded images were processed using Bruker NanoScope Analysis software. The diffuse reflectance spectra of the samples were taken with a Shimadzu UV–vis–NIR 3600 spectrophotometer equipped with an integrating sphere attachment. The ζ-potential of the nanosheets was measured using a Malvern Zetasizer Nano ZS. The morphological features of the samples were characterized using a ZEISS Ultraplus field emission scanning electron microscope. A Micromeritics ASAP 2020 system was employed to determine the surface area of the samples using the data acquired through the Brunauer–Emmett–Teller (BET) method at 77 K. X-ray photoelectron spectroscopy (XPS) was performed using a Thermo Scientific K-α spectrometer (Thermo Fisher Scientific, Waltham, MA) with a monochromatized Al Kα X-ray source (spot size ∼ 400 μm) to examine the elemental composition and to estimate valance band values.

### 2.4 Photocatalytic Characterization

#### 2.4.1 Rhodamine B Degradation

Since both the parent Bi_2_SrTa_2_O_9_ structure and restacked nanosheets have a band gap in the UV range (Figure S2), we used the full solar spectrum for the hydrogen evolution test and a UV-C lamp (254 nm) for the degradation of RhB. In the case of Rhodamine B degradation, 50 mg of the photocatalyst was added to 50 mL of RhB solution (10^–5^ M) under vigorous stirring for 30 min in dark medium until reaching equilibrium adsorption–desorption conditions. Afterward, the suspension was irradiated with 254 nm UV light at 25 °C under constant stirring. The suspension is placed in a quartz beaker at a distance of 10 cm away from the light source, where the light intensity is about 17 mW/cm^2^. To investigate the dye degradation (discoloration), 3 mL of the suspension was collected using a micropipette at the given time intervals and centrifuged, and the characteristic 554 nm absorption wavelength of RhB was monitored with a UV–vis light spectrometer. UV–vis diffuse reflectance spectra of the samples were taken with a Shimadzu UV–vis–NIR 3600 spectrophotometer with an integrating sphere attachment. To test the stability of the photocatalytic performance of the layered perovskites, the nanosheets were reused three times after being recycled by the following procedure. After each photocatalytic experiment, the nanosheets are separated from the suspension by centrifugation and dried at 80 °C for 3 h. To compensate for the loss of the photocatalyst during the separation and drying, another set of experiments was carried out on extra batches of suspension and each of them was exposed to a UV source for the same amount of time and then dried to be used later.

#### 2.4.2 Hydrogen Evolution

The hydrogen evolution reaction (HER) experiments were carried out in 10 mL quartz cells using a xenon lamp (300 W, Newport, 66901) as the light source with an A.M. 1.5 filter. Ten milligrams of the photocatalyst was added into 5 mL of an aqueous solution containing 10 vol % methanol as the hole scavenger. The solution was stirred during the test to homogenize the catalyst, and no cocatalyst was used in the experiments. A cooling water bath was used to control the temperature and prevent any thermal effect during the experiment. Before starting the experiments, the solution was saturated with argon for 30 min to eliminate the dissolved oxygen under dark conditions, and the cell was sealed with two septa to isolate it from the air. Argon gas was continuously passed between the two septa during the experiment, preventing any possible gas leakage or air contamination.

A gas chromatogram (GC-2014 Shimadzu, equipped with a thermal conductivity detector (TCD), argon as a carrier gas, and 5 Å molecular sieve column) was used to determine the H_2_ gas quantity. A sample of the gas (5 mL) in the headspace was taken using a 100 μL gas-tight syringe during the reaction, and it was then injected into the gas chromatograph per hour. Transient photocurrent experiments were performed on the films using a chronoamperometric technique. All experiments were carried out in a three-electrode cell and K_2_SO_4_ electrolyte (0.5 M) in the presence of methanol as a hole scavenger. The working, counter, and reference electrodes were FTO-coated with the prepared powder, a Pt wire, and Calomel (Sat’ed KCl) electrodes, respectively. Photocurrent responses were obtained at 1.2 V (vs SCE) under chopped light (300 W xenon light source).

## 3 Results and Discussion

### 3.1 Phase and Morphology

**Figure 2:**
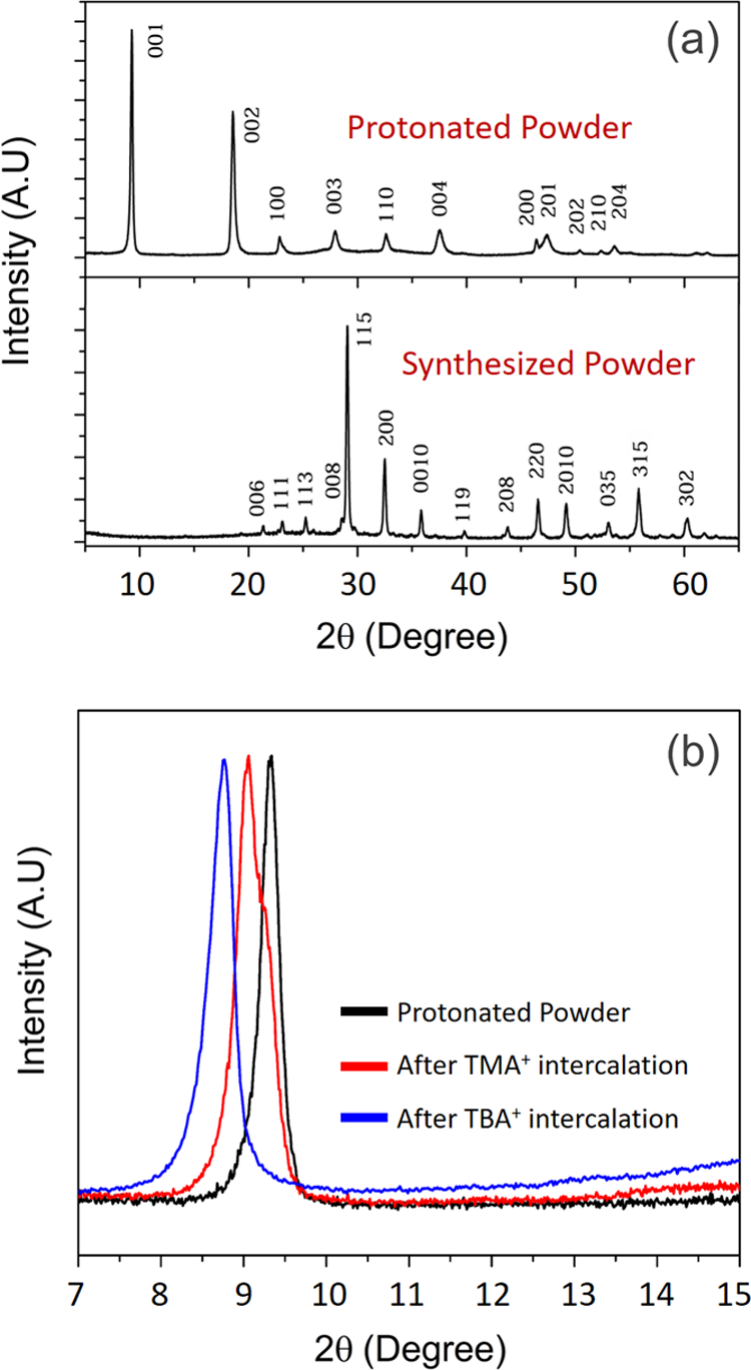
XRD patterns of the double-layered bismuth strontium tantalite and the protonate powder (a). Comparison of the normalized diffraction pattern of protonated powder with TMA^+^-and TBA^+^-intercalated samples clearly shows the shift toward a low 2θ angle in the (001) plane (b).

**Figure 3:**
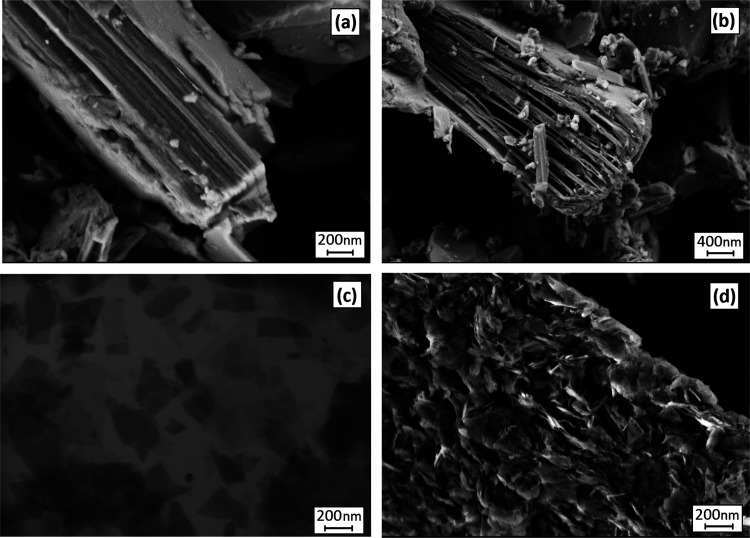
SEM image of (a) synthesized Bi_2_SrTa_2_O_9_ powder with the layered structure, (b) partially exfoliated nanoparticles after the protonation step and selective leaching of [Bi_2_O_2_]^2+^ interlayers, (c) SEM image of the obtained [SrTa_2_O_7_]^2–^ nanosheets after the step, and (d) image of the restacked nanosheets.

**Figure 4:**
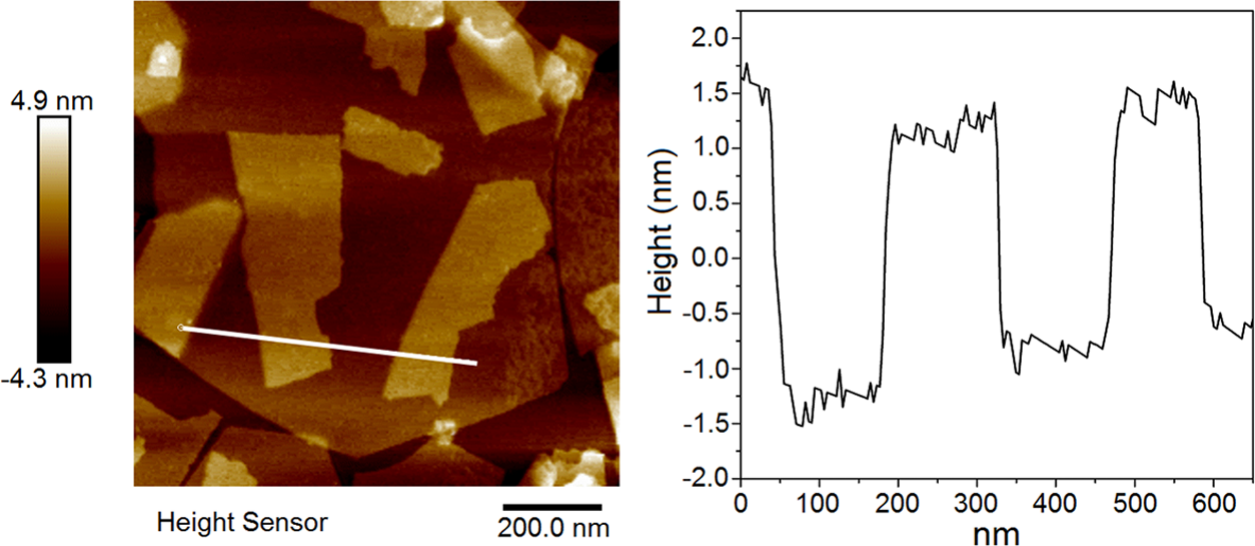
AFM image of the obtained [SrTa_2_O_7_]^2–^ nanosheets on the mica.

According to the XRD spectra presented in Figure 2a, successful synthesis of single-phase Bi_2_SrTa_2_O_9_ (BST) can be confirmed. All the diffraction peaks are perfectly indexed with the standard data of the orthorhombic BST phase (PDF 00-049-0609) with no impurity or secondary phase. In addition, the XRD pattern related to the acid-washed powder reveals the selective leaching of the [Bi_2_O_2_]^2+^ layers and simultaneous protonation of the structure.^41^ To track the intercalation steps, the strongest peak, related to the (001) crystallographic plane, was selected as the criterion, since it is a diffraction from layers stacked in the c-direction. Based on the results presented in Figure 2b, there is a shift in the (001) diffraction peak toward lower 2θ angles by the intercalation of TMA^+^ and TBA^+^ compared to the protonated powder. This shift stems from the increase in the layer distance in the stacking direction due to the entrancement of large species (TMA^+^ and TBA^+^ ions) within the structure. The yield of exfoliation increased especially upon using TMA^+^ as the intermediate step, since the cation size of TMA^+^ (0.50–0.60 nm) is higher than that of ethylamine and lower than that of TBA^+^ (0.95–1.05 nm).^42^ During the intercalation process, a gradual increase in the interlayer distance could prevent the sheets from breaking by reducing the sudden tension between the sheets caused by intercalation of large molecules.^43^ As a result, a gradual increase in the interlayer distance in three steps can lower the chance of fracture of the sheets and enhance the chance of obtaining bigger nanosheets. It should be pointed out that the XRF results (Supportive Information, Table S1) indicate that the level of bismuth in the restacked nanosheets (3.6%) is significantly lower than that of the as-synthesized powder (46.6%), which confirms the effective leaching of [Bi_2_O_2_]^2+^ layers. The XPS data also confirm the presence of Bi^3+^ ions inside the structure of the restacked nanosheets (Figure S3). Since Bi was observed even for the exfoliated nanosheets, the presence of Bi in the restacked nanosheet structure can be ascribed to the cation disorder (cation site mixing). The first study on cation disorder in the double-layered Aurivillius structure was conducted by Blake et al., who showed that the value of the disorder can even reach up to 13.4% depending on the size of the cation.^44^ Similar research on Bi_2_SrTa_2_O_9_ showed that Bi ions are capable of occupying [A] sites belonging to cations such as Sr^2+^ in [A*_n_*_–1_B*_n_*O_3*n*+1_]^2–^ nanosheets. This resulted in a significant level of cation disorder, which depends not only on the nature of the cations but also on the thermal treatment of the samples during the synthesis.^45^ It should be noted that cation disorder is not uncommon in the layered-type materials and a similar phenomenon has also been reported in the case of triple- and quadro-layered perovskites.^46−49^ The morphological features of the as-synthesized powder and the partially exfoliated nanostructure are presented in Figure 3a and b, respectively. The removal of the interlayer [Bi_2_O_2_]^2+^ ions after the proton exchange reaction opens up the layers as seen in Figure 3b. The STEM image in Figure 3c and SEM image in Figure 3d reveal that the layered Aurivillius phase was successfully exfoliated to single 2D nanosheets, which were restacked with high surface area. The AFM measurement confirms the fabrication of unilamellar 2D nanosheets with an average thickness of 2 nm and varied lateral dimensions (Figure 4). As can be seen in Figure 4, smaller nanosheets were arranged on top of larger nanosheets.The BET surface area measurement shows drastic morphological change induced by protonation and exfoliation steps. For the as-synthesized powder, the specific surface area is found to be 0.91 m^2^/g, while for the protonated powder and restacked nanosheets, this value is increased to 2.70 and 13.25 m^2^/g, respectively. It is worthy of note that the surface area of the Aurivillius-type structure synthesized via solid-state reactions is typically lower than <1 m^2^/g^50,51^ and it can be enhanced to 2–5 m^2^/g by manipulating the morphology (flower-like and sponge-like structures) or doping.^52,53^ Recently, Majumdar et al.^54^ have reported a surface area of 15 m^2^/g for the intact Aurivillius-type layered structure (with [Bi_2_O_2_]^2+^ interlayers), which is very close to that of the restacked single nanosheets obtained in the present study (13.25 m^2^/g). However, the nanosheets obtained in this work are extremely thin, measuring at only a few nanometers, and lack [Bi_2_O_2_]^2+^ interlayers in their structure.

### 3.2 Photocatalytic Activity

#### 3.2.1 Rhodamine B Degradation

**Figure 5:**
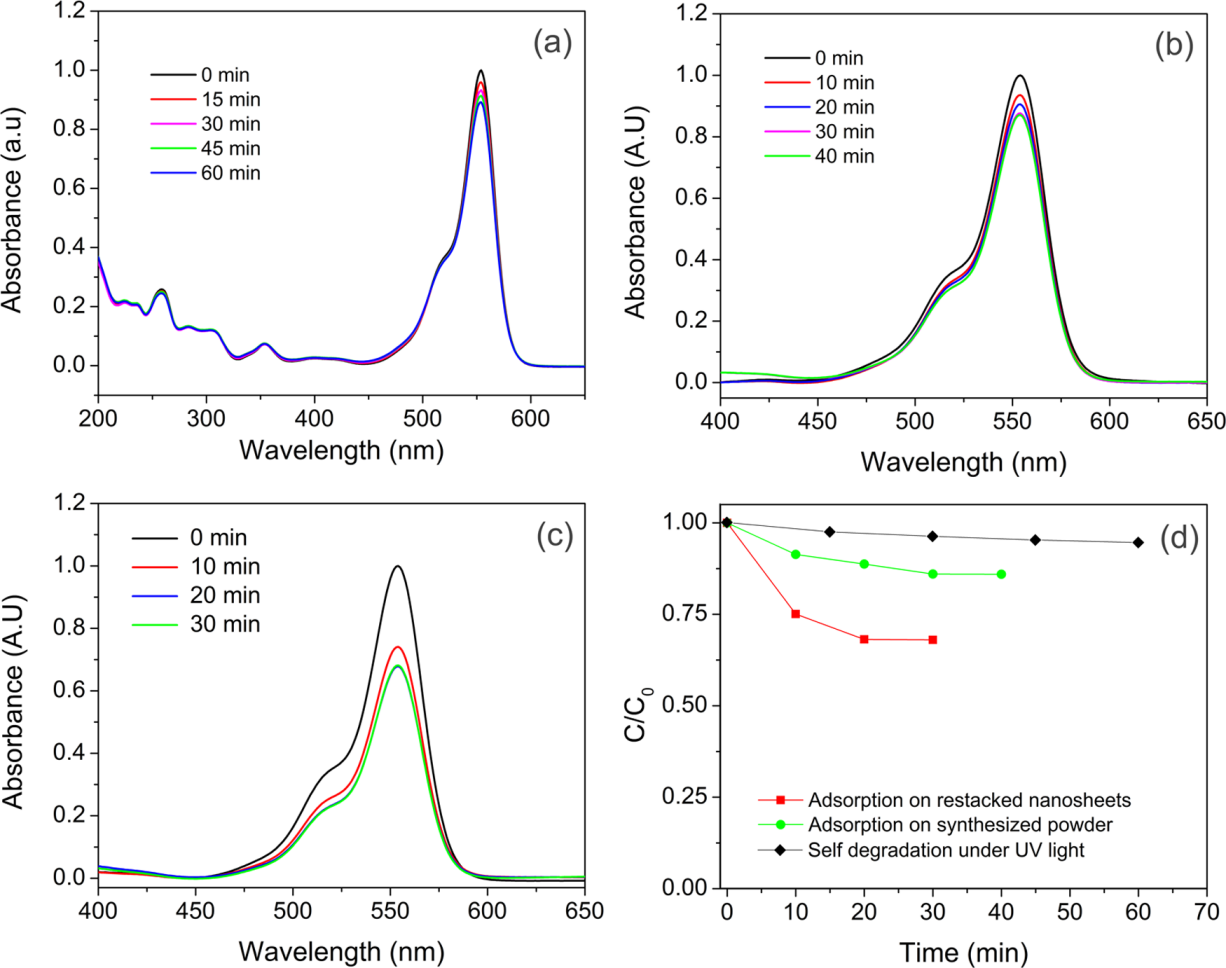
Temporal UV–vis spectra changes of RhB for (a) blank solution under 254 nm UV light, (b) synthesized powder–dye suspension in the dark, (c) restacked nanosheet–dye suspension in the dark, and (d) RhB self-degradation under UV/adsorption in the dark versus time.

**Figure 6:**
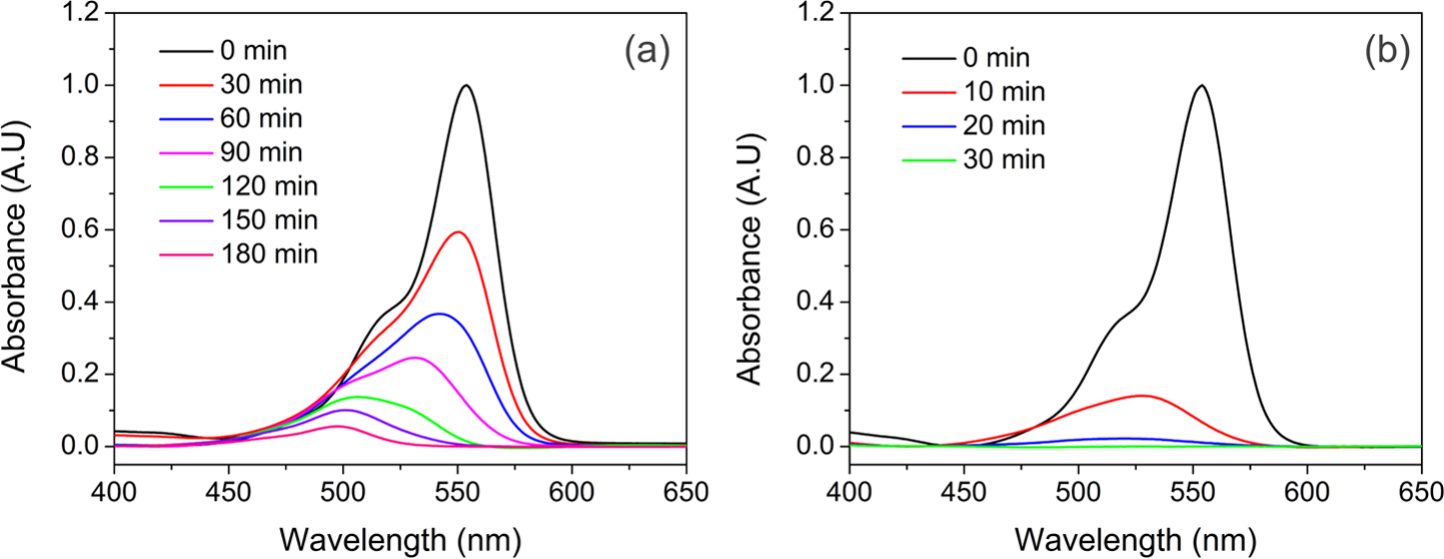
Temporal UV–vis spectral change for the RhB solution during photocatalytic degradation by (a) synthesized Bi_2_SrTa_2_O_9_ powder and (b) restacked [SrTa_2_O_7_]^2–^ nanosheets.

**Figure 7:**
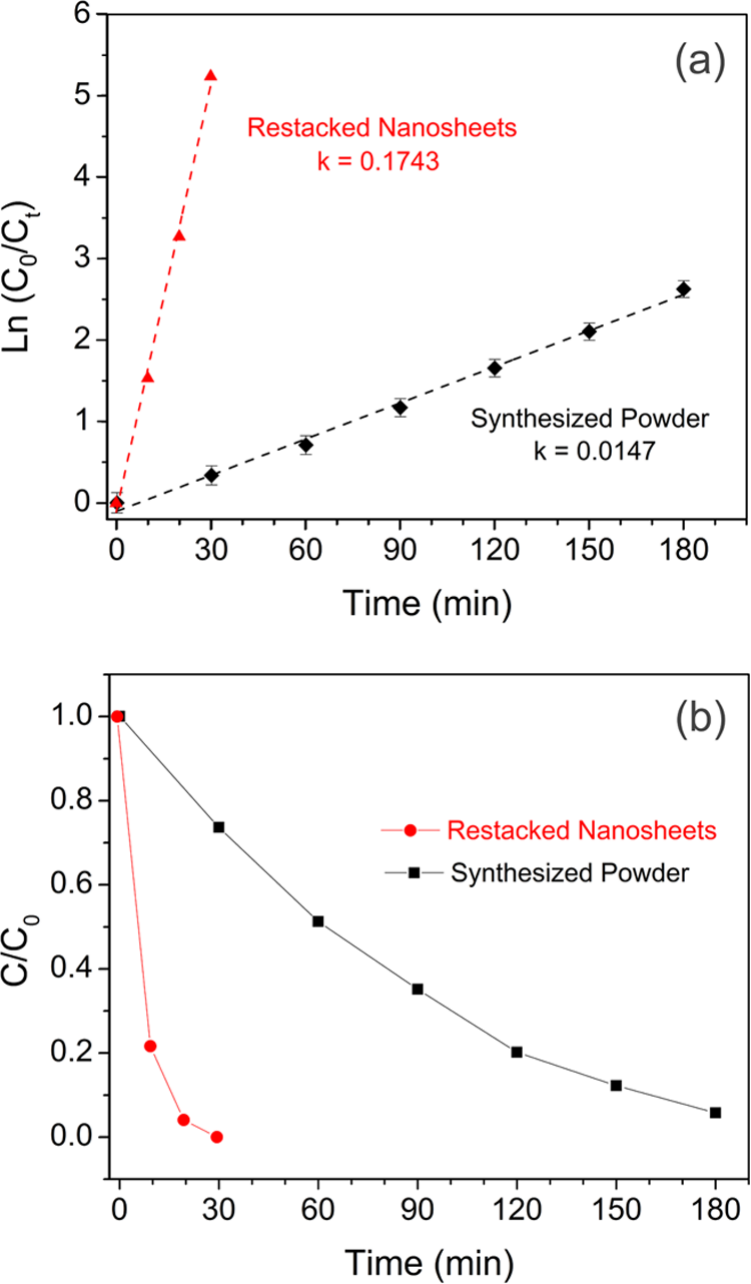
Plot of ln(*C*_0_/*C_t_*) vs irradiation time for obtaining the reaction rate constant (a) and trend of the RhB degradation by the synthesized powder and restacked nanosheets as a function of irradiation time (b).

**Figure 8:**
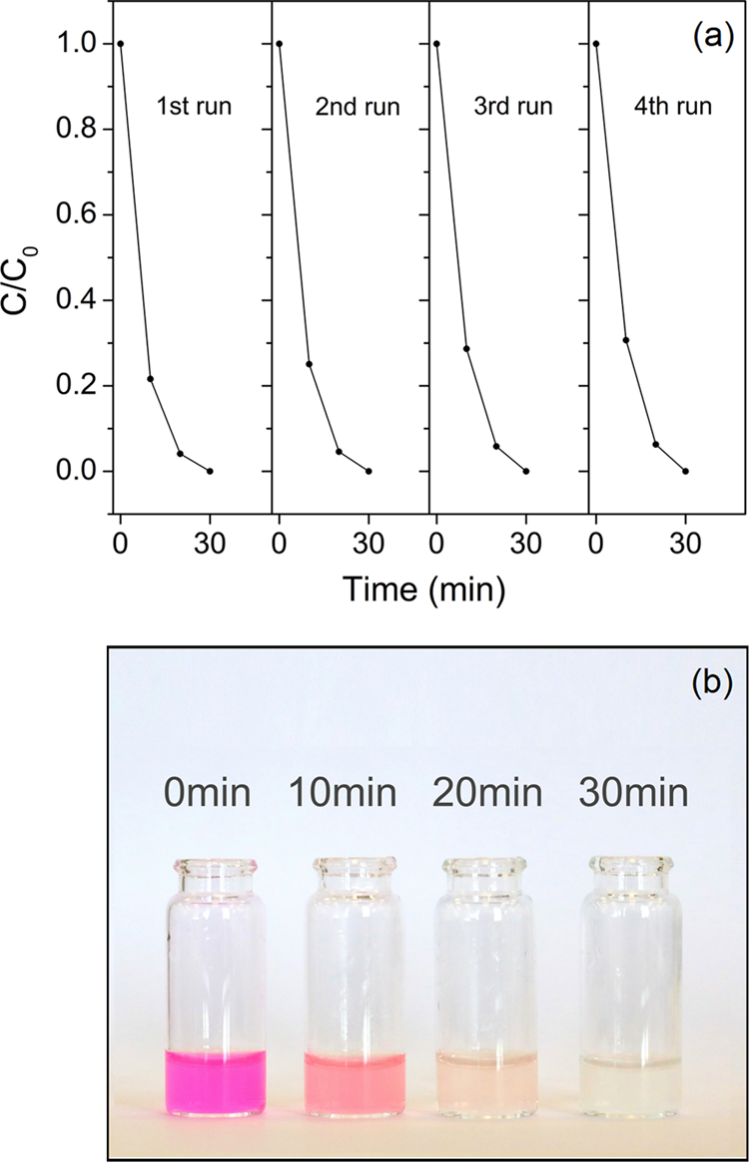
Cyclic photodegradation of RhB over the restacked nanosheets under UV–vis light irradiation (a) and visual comparison for the degradation of Rhodamine B over time for samples containing restacked nanosheets (b).

To examine the degradation capability of the as-synthesized powder with restacked nanosheets, with respect to RhB, UV–vis absorption spectroscopy was conducted as described in Section 2.4.1. In addition, RhB degradation was monitored in the absence of any photocatalysts with 1 h of UV lamp irradiation (distance: 10 cm, power: 17 mW/cm^2^). Spectroscopic data reveal that RhB solution shows less than 5% self-degradation under a 254 nm light source after 60 min (Figure 5a). To compare the adsorption capability of the synthesized powder with that of the restacked nanosheets, a photocatalyst–dye suspension of both samples was mixed in the dark. As shown in Figure 5b,c, the collected UV–vis absorption spectra of the aqueous solution at 10 min intervals verify that adsorption–desorption of the dye on the surface of the catalyst reaches the equilibrium state after 30 and 20 min for the as-synthesized nanoparticles and restacked nanosheets, respectively. The rate of self-degradation of RhB under UV light and also RhB adsorption on [SrTa_2_O_7_]^2–^ restacked nanosheets and Bi_2_SrTa_2_O_9_ powder are illustrated in Figure 5d, in which [*C*_0_] is the initial concentration of the dye and [*C*] is the measured concentration at a given time. These data show that the restacked nanosheets with the H_2_SrTa_2_O_7_ formula have almost 3 times higher adsorption capacity in comparison with that of Bi_2_SrTa_2_O_9_ nanoparticles. This enhancement in the adsorption capacity would be attributed to the higher surface area of the nanosheets as well as the electrostatic attractive interaction between the negatively charged surface of the nanosheets (ζ-potential = −44 mV) and the positively charged diethylamine functional groups of the RhB (Supporting Information, Figure S4).^55,56^ It was also reported that electron transfer is more efficient in catalysts with strong Lewis acidic nature since it can provide stronger interactions between the dye and the catalyst^57^ and lower recombination rate of electrons and holes by facilitating the interfacial charge transfer process.^58^ After equilibrium is reached, the photocatalyst–dye suspension is exposed to a light source, and the change in both the intensity and position of the absorbance peak of RhB is monitored to study the extent of the degradation. In the case of the as-synthesized powder (Figure 6), the temporal evolution of UV–vis absorption spectra indicates that continuous reduction in the intensity of the absorbance peak (hypochromic shift) is accompanied by the peak shift to a lower wavelength (hypsochromic shift), while the whole process takes 180 min to completed. However, in the case of the restacked nanosheets, degradation of RhB is conducted in less than 30 min, which also results in a change in both the intensity and position. According to the literature, these two behaviors can be assigned to two different competitive decomposition mechanisms.^55,59−62^ Reduction in the absorption intensity is related to the dissociation of the conjugated structure and aromatic chromophore group, which is responsible for the color of the dye. However, the peak shift is attributed to the stepwise *N*-de-ethylation process and formation of the series of *N*-de-ethylated intermediates. Each of these intermediates has its absorption peak in the visible region. In the case of the tetraethylated RhB, the maximum absorption peak appears at 554 nm. However, this value decreases to 539, 522, and 510 nm for tri, di-, monoethylated RhB, respectively, and eventually reaches 498 nm for nonethylated Rhodamine.^63,64^ Experimental results show that for both samples, these two processes occur simultaneously. In the case of the as-synthesized powder, *N*-de-ethylation and aromatic chromophore destruction are completed roughly at the same time, whereas in the case of the restacked nanosheets, full decoloration of RhB takes place prior to full *N*-de-ethylation, even though the rate of both reactions has increased. The kinetics of the photocatalytic RhB degradation over our catalysts in water can be described according to the pseudo-first-order equation as given below^65^
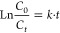
1where [*C*_0_] is the initial concentration of the dye, [*C*_*t*_] is the measured concentration at a given time, [*t*] is the time, and [*k*] is the pseudo-first-order rate constant. The model is established to describe the dependence of the reaction rate on the initial solute concentrations. Since the concentration (*C*) of RhB has a linear relation with the absorbance (*A*), the equation can also be expressed as follows
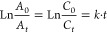
2As depicted in Figure 7, the linear relation between ln(*C*_0_/*C*) and irradiation time (*t*) indicates that the degradation of RhB follows first-order kinetics. The value of the rate constant (*k*) is calculated from the slope of the plot in Figure 7a, and it can be seen that the restacked nanosheets have one order of magnitude higher rate constant in comparison to that of the synthesized powder. Photocatalytic degradation of RhB with time by [SrTa_2_O_7_]^2–^ restacked nanosheets and Bi_2_SrTa_2_O_9_ powder is shown in Figure 7b. The photodegradation efficiency (PE) for each experiment is calculated using the following equation
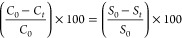
3*S*_0_ is the integrated area of the peak at the initial concentration and *S_t_* is the integrated area of the peak at the given time. Notably, the PE value for the [SrTa_2_O_7_]^2–^ restacked nanosheets reached as high as ∼100% in 30 min, which is about 4 times faster than that of the Bi_2_SrTa_2_O_9_ (PE = 26%) in the same illumination time. Better photocatalytic properties of the restacked nanosheets can be attributed not only to higher surface area and higher adsorption capacity but also to shortened distance for photogenerated electrons and holes induced by particle size reduction. The stability and reusability of the restacked nanosheets, as the photocatalyst, were evaluated after four successive same cycles (Figure 8), and it was found that there is no significant change in the efficiency of the photocatalyst.

#### 3.2.2 Hydrogen Evolution

**Figure 9:**
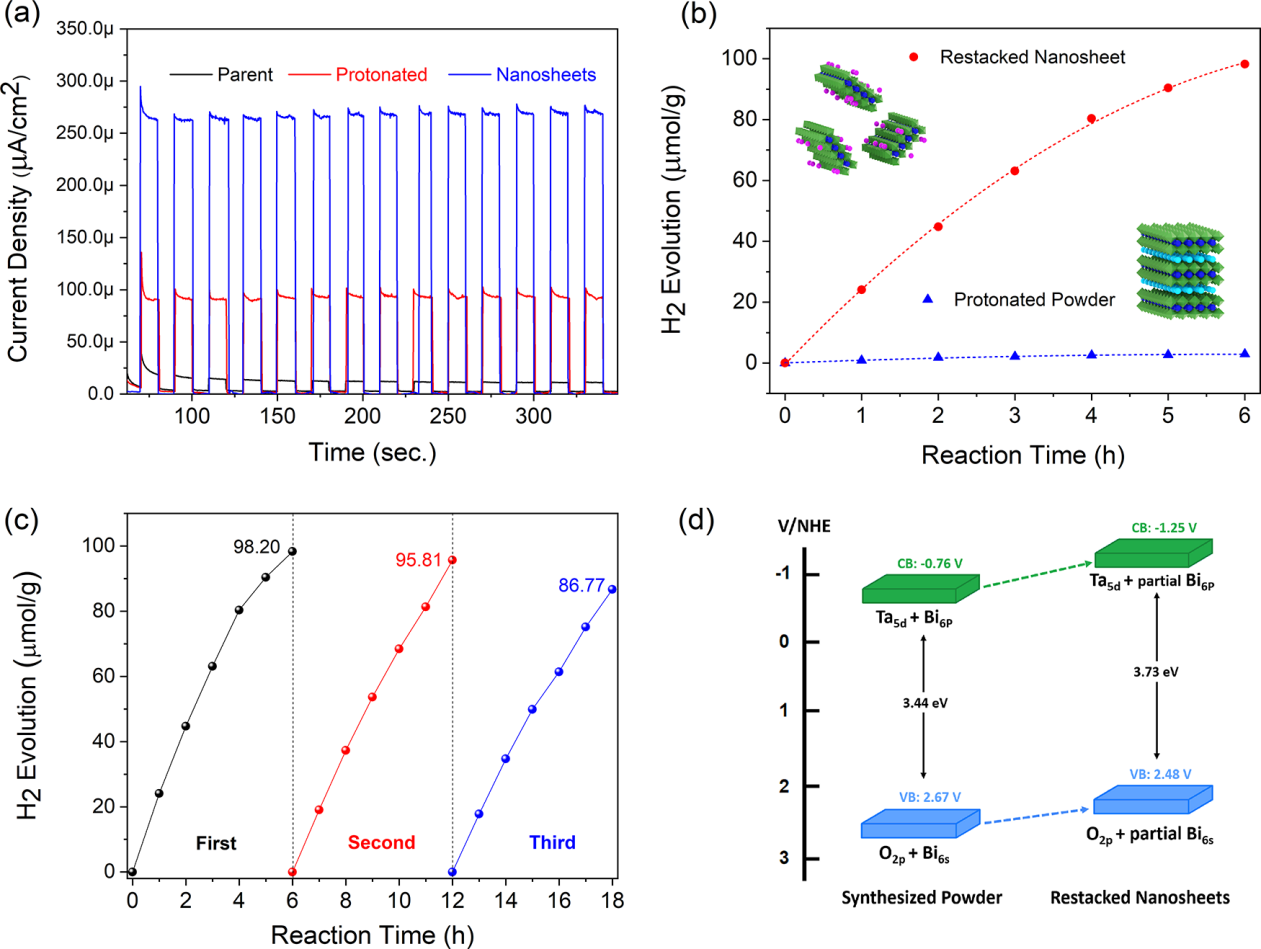
Photocurrent densities of coated films under 1.2 V vs SCE bias (a), time courses of H_2_ evolution of protonated powder and restacked nanosheets by UV photoirradiation (b), repeated time courses of photocatalytic hydrogen production for nanosheets under UV light irradiation in methanol aqueous solution (10 vol %) (c), and schematic of the band structure for the synthesized powder and restacked nanosheets (d).

We have tested the samples for the photocatalytic hydrogen evolution reaction since the dye degradation reaction proved that the nanosheets showed photocatalytic activity. The photoelectrochemical response of the samples was tested with transition photocurrent experiments. As can be observed in Figure 9a, the parent structure, the proton-exchanged form, and the exfoliated 2D perovskite nanosheets produced photocurrent under the full light spectrum. The current densities of a parent, protonated powder, and 2D nanosheets were 15, 100, and 260 μA/cm^2^ at 1.2 V vs SCE, respectively. As shown in the figure, 2D nanosheets obtained from exfoliation of the layered perovskite showed dramatically enhanced photocurrent compared to that of the parent and proton-exchanged powders, which is consistent with the data in the literature.^66,67^ In comparison to the restacked nanosheets, the parent and protonated powders showed lower photocurrent due to the longer diffusion length for electron–hole pairs in layered structures, which increases the recombination rate, thus reducing efficiency.^67,68^ The higher photocurrent of the 2D nanosheets can be attributed to their higher specific surface area and shorter and faster charge transfer pathways.^69^

Figure 9b shows the results of photocatalytic H_2_ evolution experiments for protonated and restacked nanosheet samples for 6 h without any cocatalyst loading. Since the nanosheets obtained from the layered Aurivillius structure showed superior photocatalytic activity against the dye degradation reaction compared to the bulk structure, we have also tested the potential of nanosheets for photocatalytic hydrogen evolution from water. The results showed that the parent layered structure was inefficient in the H_2_ evolution reaction. On the contrary, the restacked nanosheets exhibited high activity for H_2_ evolution under UV light irradiation. At the end of 6 h, the amounts of H_2_ obtained from nanosheets and protonated powders were 98.20 and 3.00 μmol g^–1^, respectively. The amount of H_2_ obtained from the restacked nanosheets was about 30 times higher than that obtained from the protonated ones. This behavior can also be attributed to the fact that nanosheet structures have a greater surface area and a higher length-to-thickness ratio than those of bulk samples. These conditions not only provide more active sites for the reduction of water to H_2_ but also improve charge carrier separation and shorten the diffusion length for photoinduced electrons and holes, reducing the risk of recombination compared to the bulk phase. Furthermore, the stability of the nanosheets for photocatalytic H_2_ evolution was examined (Figure 9c). The solution was saturated with argon every 6 h to remove any H_2_ from the cell. The stability of the photocatalyst was tested in an 18 h experiment. Comparing the stability cycles, the rate of H_2_ evolution after the third cycle is about 90% of that in the first cycle. As a result of the stability test, it was observed that the high activity obtained from the nanosheets was reproducible and highly stable.

The obtained optical properties of the samples are consistent with the photocatalytic hydrogen evolution results as given below. Generally, bismuth-based oxides have become one of the attractive candidates due to the high activity under visible light, which is correlated to the d^10^s^2^ electronic structure of Bi and dispersed hybridized valence band (V.B) containing Bi 6s and O 2p orbitals, which leads to high mobility of holes.^70−72^ In the case of Bi_2_SrTa_2_O_9_, both Ta 5d and Bi 6p states have contribution in forming the conduction band (C.B). Since the Bi 6p state has lower energy than that of the Ta 5d state, an increase in the band gap energy after the removal of Bi^3+^ for protonated and restacked nanosheets was expected, which was also confirmed by UV–visible results (Figure S2). Combination of data from valence band measurements by XPS (Figure S5) with UV–vis data approves that the positions of both the V.B and C.B in the case of the restacked nanosheets are shifted to more negative potentials vs S.H.E. due to the removal of Bi^3+^ from the interlayer as shown in Figure 9d. Even though the band gap is increased for the protonated powder and restacked nanosheets, this change in the electronic structure is favorable for the hydrogen evolution reaction due to the more negative C.B. potential vs H^+^/H_2_ reduction potential.

## 4 Conclusions

In conclusion, pure Bi_2_SrTa_2_O_9_ nanoparticles were synthesized through a solid-state reaction, and a colloidal suspension of [SrTa_2_O_7_]^2–^ nanosheets was obtained through a protonation and three-step liquid exfoliation process. These nanosheets are useful in photocatalytic reactions due to their high surface area and short charge transport pathways. The exfoliation process was found to increase the specific surface area of the nanosheets by a factor of 13, leading to a higher active area for photocatalytic reactions. The photocatalytic activity of the samples was evaluated through hydrogen evolution and degradation of RhB in an aqueous solution. The stability of the exfoliated nanosheets was found to be higher than that of the parent structure for both hydrogen evolution and Rhodamine B removal. The negative charge of the exfoliated nanosheets was found to increase their dye adsorption capacity. Rhodamine molecules were adsorbed onto the surface of the nanosheets through their positively charged diethylamine functional group. It was also observed that exfoliation not only increases the rate of photocatalytic reactions by providing more adsorption sites but also increases the rate of photocatalytic decomposition of the dye through stronger interactions between the dye and the catalyst. These results suggest that the fabrication of strong acid solids could be a promising method for efficient cationic pollutant adsorption and the transfer of photoinduced electrons. Additionally, optical characterization showed that the position of the conduction band in these samples was shifted to a more negative potential, which is beneficial for photocatalytic H_2_ production.

## Supporting Information Available

Composition of the synthesized powders analyzed using XRF and UV–visible spectra with Tauc plots, XPS valence band spectra of the restacked nanosheets and synthesized powder, and possible adsorption of Rhodamine B on negatively charged nanosheets (PDF)

## References

[ref1] Fujishima A.; Honda K. Electrochemical Photolysis of Water at a Semiconductor Electrode. Nature 1972, 238, 37–38. 10.1038/238037a0. 12635268

[ref2] Yamaguchi Y.; Usuki S.; Yamatoya K.; Suzuki N.; Katsumata K. I.; Terashima C.; Fujishima A.; Kudo A.; Nakata K. Efficient Photocatalytic Degradation of Gaseous Acetaldehyde over Ground Rh-Sb Co-Doped SrTiO_3_ under Visible Light Irradiation. RSC Adv. 2018, 8, 5331–5337. 10.1039/c7ra11337d. 35542434 PMC9078108

[ref3] Wang C. Y.; Zhang Y. J.; Wang W. K.; Pei D. N.; Huang G. X.; Chen J. J.; Zhang X.; Yu H. Q. Enhanced Photocatalytic Degradation of Bisphenol A by Co-Doped BiOCl Nanosheets under Visible Light Irradiation. Appl. Catal., B 2018, 221, 320–328. 10.1016/j.apcatb.2017.09.036.

[ref4] Dai Z.; Qin F.; Zhao H.; Ding J.; Liu Y.; Chen R. Crystal Defect Engineering of Aurivillius Bi_2_MoO_6_ by Ce Doping for Increased Reactive Species Production in Photocatalysis. ACS Catal. 2016, 6, 3180–3192. 10.1021/acscatal.6b00490.

[ref5] Malik R.; Tomer V. K. State-of-the-Art Review of Morphological Advancements in Graphitic Carbon Nitride (g-CN) for Sustainable Hydrogen Production. Renewable Sustainable Energy Rev. 2021, 135, 110235 10.1016/j.rser.2020.110235.

[ref6] Karthikeyan C.; Arunachalam P.; Ramachandran K.; Al-Mayouf A. M.; Karuppuchamy S. Recent Advances in Semiconductor Metal Oxides with Enhanced Methods for Solar Photocatalytic Applications. J. Alloys Compd. 2020, 828, 154281 10.1016/j.jallcom.2020.154281.

[ref7] Zhang C.; Kuang D. Bin.; Wu W. Q. A Review of Diverse Halide Perovskite Morphologies for Efficient Optoelectronic Applications. Small Methods 2020, 4, 1900662 10.1002/smtd.201900662.

[ref8] Ida S.; Ishihara T. Recent Progress in Two-Dimensional Oxide Photocatalysts for Water Splitting. J. Phys. Chem. Lett. 2014, 5, 2533–2542. 10.1021/jz5010957. 26277941

[ref9] Dariani R. S.; Esmaeili A.; Mortezaali A.; Dehghanpour S. Photocatalytic Reaction and Degradation of Methylene Blue on TiO_2_ Nano-Sized Particles. Optik 2016, 127, 7143–7154. 10.1016/j.ijleo.2016.04.026.

[ref10] Kumar V.; Choudhary S.; Malik V.; Nagarajan R.; Kandasami A.; Subramanian A. Enhancement in Photocatalytic Activity of SrTiO_3_ by Tailoring Particle Size and Defects. Phys. Status Solidi A 2019, 216, 1900294 10.1002/pssa.201900294.

[ref11] Yang G.; Chen D.; Ding H.; Feng J.; Zhang J. Z.; Zhu Y.; Hamid S.; Bahnemann D. W. Well-Designed 3D ZnIn_2_S_4_ Nanosheets/TiO_2_ Nanobelts as Direct Z-Scheme Photocatalysts for CO_2_ Photoreduction into Renewable Hydrocarbon Fuel with High Efficiency. Appl. Catal., B 2017, 219, 611–618. 10.1016/j.apcatb.2017.08.016.

[ref12] Hao R.; Wang G.; Jiang C.; Tang H.; Xu Q. In Situ Hydrothermal Synthesis of g-C_3_N_4_/TiO_2_ Heterojunction Photocatalysts with High Specific Surface Area for Rhodamine B Degradation. Appl. Surf. Sci. 2017, 411, 400–410. 10.1016/j.apsusc.2017.03.197.

[ref13] Yang H.; Zhou Y.; Wang Y.; Hu S.; Wang B.; Liao Q.; Li H.; Bao J.; Ge G.; Jia S. Three-Dimensional Flower-like Phosphorus-Doped g-C_3_N_4_ with a High Surface Area for Visible-Light Photocatalytic Hydrogen Evolution. J. Mater. Chem. A 2018, 6, 16485–16494. 10.1039/c8ta05723k.

[ref14] Lu X.; Xu K.; Chen P.; Jia K.; Liu S.; Wu C. Facile One Step Method Realizing Scalable Production of g-C_3_N_4_ Nanosheets and Study of Their Photocatalytic H_2_ Evolution Activity. J. Mater. Chem. A 2014, 2, 18924–18928. 10.1039/c4ta04487h.

[ref15] Zhang X.; Qin J.; Xue Y.; Yu P.; Zhang B.; Wang L.; Liu R. Effect of Aspect Ratio and Surface Defects on the Photocatalytic Activity of ZnO Nanorods. Sci. Rep. 2014, 4, 4596 10.1038/srep04596. 24699790 PMC3975220

[ref16] Majumdar A.; Pal A. Optimized Synthesis of Bi_4_NbO_8_Cl Perovskite Nanosheets for Enhanced Visible Light Assisted Photocatalytic Degradation of Tetracycline Antibiotics. J. Environ. Chem. Eng. 2020, 8, 103645 10.1016/j.jece.2019.103645.

[ref17] Chen W.; Li C.; Gao H.; Yuan J.; Shangguan W.; Su J.; Sun Y. Photocatalytic Water Splitting on Protonated Form of Layered Perovskites K_0.5_La_0.5_Bi_2_M_2_O_9_ (M = Ta; Nb) by Ion-Exchange. Int. J. Hydrogen Energy 2012, 37, 12846–12851. 10.1016/j.ijhydene.2012.05.090.

[ref18] Miseki Y.; Kato H.; Kudo A. Water Splitting into H_2_ and O_2_ over Niobate and Titanate Photocatalysts with (111) Plane-Type Layered Perovskite Structure. Energy Environ. Sci. 2009, 2, 306–314. 10.1039/b818922f.

[ref19] Kalantar-zadeh K.; Ou J. Z.; Daeneke T.; Mitchell A.; Sasaki T.; Fuhrer M. S. Two Dimensional and Layered Transition Metal Oxides. Appl. Mater. Today. 2016, 5, 73–89. 10.1016/j.apmt.2016.09.012.

[ref20] Osada M.; Sasaki T. Exfoliated Oxide Nanosheets: New Solution to Nanoelectronics. J. Mater. Chem. 2009, 19, 2503–2511. 10.1039/b820160a.

[ref21] Chevallier V.; Nihoul G.; Madigou V. Exfoliated Nanoplatelets of an Aurivillius Phase, Bi_3.25_La_0.75_Ti_3_O_12_: Characterisation by X-Ray Diffraction and by High-Resolution Electron Microscopy. J. Solid State Chem. 2008, 181, 439–449. 10.1016/j.jssc.2007.12.012.

[ref22] Ida S.; Ogata C.; Unal U.; Izawa K.; Inoue T.; Altuntasoglu O.; Matsumoto Y. Preparation of a Blue Luminescent Nanosheet Derived from Layered Perovskite Bi_2_SrTa_2_O_9_. J. Am. Chem. Soc. 2007, 129, 8956–8957. 10.1021/ja073105b. 17602633

[ref23] Kim J. Y.; Chung I.; Choy J. H.; Park G. S. Macromolecular Nanoplatelet of Aurivillius-Type Layered Perovskite Oxide, Bi_4_Ti_3_O_12_. Chem. Mater. 2001, 13, 2759–2761. 10.1021/cm0102436.

[ref24] Silyukov O. I.; Kurnosenko S. A.; Zvereva I. A. Intercalation of Methylamine into the Protonated Forms of Layered Perovskite-Like Oxides HLnTiO_4_ (Ln = La and Nd). Glass Phys. Chem. 2018, 44, 428–432. 10.1134/S1087659618050176.

[ref25] Nicolosi V.; Chhowalla M.; Kanatzidis M. G.; Strano M. S.; Coleman J. N. Liquid Exfoliation of Layered Materials. Science 2013, 340, 1226419 10.1126/science.1226419.

[ref26] Deng M.; Ye M.; Li T.; Huang H.; Yuan W. X.; Jiang H.; Lin P.; Zeng X.; Ke S. Synthesis of Ferroelectric KNbO_3_ nanosheets by Liquid Exfoliation of Layered Perovskite K_2_NbO_3_F. J. Alloys Compd. 2017, 698, 357–363. 10.1016/j.jallcom.2016.11.209.

[ref27] Phonsuksawang P.; Waehayee A.; Jiamprasertboon A.; Nijpanich S.; Siritanon T. Facile and Rapid Exfoliation of HTiNbO_5_ Nanosheets for Photocatalytic Applications. Mater. Lett. 2022, 323, 132492 10.1016/J.MATLET.2022.132492.

[ref28] Wei Y. B.; Guo X. J.; Li B. J. Exfoliation-Co-Flocculation Fabrication of Novel Porous HTiNbO_5_/Reduced Graphene Oxide Nanocomposites and the Photocatalytic Performance. Microporous Mesoporous Mater. 2019, 287, 144–151. 10.1016/J.MICROMESO.2019.06.001.

[ref29] Hsu C.-W.; Awaya K.; Tsushida M.; Miyano T.; Koinuma M.; Ida S. Water Splitting Using a Photocatalyst with Single-Atom Reaction Sites. J. Phys. Chem. C 2020, 124, 20846–20853. 10.1021/acs.jpcc.0c03132.

[ref30] Ebina Y.; Sakai N.; Sasaki T. Photocatalyst of Lamellar Aggregates of RuOx-Loaded Perovskite Nanosheets for Overall Water Splitting. J. Phys. Chem. B 2005, 109, 17212–17216. 10.1021/jp051823j. 16853196

[ref31] Maeda K.; Mallouk T. E. Comparison of Two- and Three-Layer Restacked Dion-Jacobson Phase Niobate Nanosheets as Catalysts for Photochemical Hydrogen Evolution. J. Mater. Chem. 2009, 19, 4813–4818. 10.1039/b903692j.

[ref32] Wang P.; Cheng M.; Zhang Z. On Different Photodecomposition Behaviors of Rhodamine B on Laponite and Montmorillonite Clay under Visible Light Irradiation. J. Saudi Chem. Soc. 2014, 18, 308–316. 10.1016/j.jscs.2013.11.006.

[ref33] Blair V. L.; Nichols E. J.; Liu J.; Misture S. T. Surface Modification of Nanosheet Oxide Photocatalysts. Appl. Surf. Sci. 2013, 268, 410–415. 10.1016/j.apsusc.2012.12.110.

[ref34] Collu D. A.; Carucci C.; Piludu M.; Parsons D. F.; Salis A. Aurivillius Oxides Nanosheets-Based Photocatalysts for Efficient Oxidation of Malachite Green Dye. Int. J. Mol. Sci. 2022, 23, 5422 10.3390/ijms23105422. 35628232 PMC9140923

[ref35] Keeney L.; Smith R. J.; Palizdar M.; Schmidt M.; Bell A. J.; Coleman J. N.; Whatmore R. W. Ferroelectric Behavior in Exfoliated 2D Aurivillius Oxide Flakes of Sub-Unit Cell Thickness. Adv. Electron. Mater. 2020, 6, 1901264 10.1002/aelm.201901264.

[ref36] Zhou Y.; Zhang Y.; Lin M.; Long J.; Zhang Z.; Lin H.; Wu J. C. S.; Wang X. Monolayered Bi2WO6 Nanosheets Mimicking Heterojunction Interface with Open Surfaces for Photocatalysis. Nat. Commun. 2015, 6, 8340 10.1038/ncomms9340. 26359212 PMC4647850

[ref37] Maeda K.; Eguchi M. Structural Effects of Two-Dimensional Perovskite Ca_2_Nb_2_TaO_10_^-^ Nanosheets for Photocatalytic Hydrogen Evolution. Catal. Sci. Technol. 2016, 6, 1064–1069. 10.1039/c5cy01246e.

[ref38] Li B.-W.; Osada M.; Ebina Y.; Akatsuka K.; Fukuda K.; Sasaki T. High Thermal Robustness of Molecularly Thin Perovskite Nanosheets and Implications for Superior Dielectric Properties. ACS Nano 2014, 8, 5449–5461. 10.1021/nn502014c. 24797417

[ref39] Unal U.; Matsumoto Y.; Tamoto N.; Koinuma M.; MacHida M.; Izawa K. Visible Light Photoelectrochemical Activity of K_4_Nb_6_O_17_ Intercalated with Photoactive Complexes by Electrostatic Self-Assembly Deposition. J. Solid State Chem. 2006, 179, 33–40. 10.1016/J.JSSC.2005.09.038.

[ref40] Matsumoto Y.; Unal U.; Kimura Y.; Ohashi S.; Izawa K. Synthesis and Photoluminescent Properties of Titanate Layered Oxides Intercalated with Lanthanide Cations by Electrostatic Self-Assembly Methods. J. Phys. Chem. B 2005, 109, 12748–12754. 10.1021/jp0517089. 16852580

[ref41] Tsunoda Y.; Shirata M.; Sugimoto W.; Liu Z.; Terasaki O.; Kuroda K.; Sugahara Y. Preparation and HREM Characterization of a Protonated Form of a Layered Perovskite Tantalate from an Aurivillius Phase Bi_2_SrTa_2_O_9_ via Acid Treatment. Inorg. Chem. 2001, 40, 5768–5771. 10.1021/ic010266m. 11681883

[ref42] Liu Z. H.; Wang Z. M.; Yang X.; Ooi K. Intercalation of Organic Ammonium Ions into Layered Graphite Oxide. Langmuir 2002, 18, 4926–4932. 10.1021/la011677i.

[ref43] Espina A.; Jaimez E.; Khainakov S. A.; et al. Synthesis of New *n*-Alkylamines Intercalation Compounds with α-Titanium Phosphate. Process Selectivity and Structural and Morphological Characterization. Chem. Mater. 1998, 10, 2490–2496. 10.1021/cm9802090.

[ref44] Blake S. M.; Falconer M. J.; McCreedy M.; Lightfoot P. Cation Disorder in Ferroelectric Aurivillius Phases of the Type Bi_2_ANb_2_O_9_ (A = Ba, Sr, Ca). J. Mater. Chem. 1997, 7, 1609–1613. 10.1039/a608059f.

[ref45] Macquart R.; Kennedy B. J.; Shimakawa Y. Cation Disorder in the Ferroelectric Oxides ABi_2_Ta_2_O_9_, A = Ca, Sr, Ba. J. Solid State Chem. 2001, 160, 174–177. 10.1006/jssc.2001.9216.

[ref46] Hyatt N. C.; Hriljac J. A.; Comyn T. P. Cation Disorder in Bi_2_Ln_2_Ti_3_O_12_ Aurivillius Phases (Ln = La, Pr, Nd and Sm). Mater. Res. Bull. 2003, 38, 837–846. 10.1016/S0025-5408(03)00032-1.

[ref47] Kennedy B. J.; Zhou Q.; Ismunandar; Kubota Y.; Kato K. Cation Disorder and Phase Transitions in the Four-Layer Ferroelectric Aurivillius Phases ABi_4_Ti_4_O_15_ (A=Ca, Sr, Ba, Pb). J. Solid State Chem. 2008, 181, 1377–1386. 10.1016/j.jssc.2008.02.015.

[ref48] Koval V.; Skorvanek I.; Viola G.; Zhang M.; Jia C.; Yan H. Crystal Chemistry and Magnetic Properties of Gd-Substituted Aurivillius-Type Bi_5_FeTi_3_O_15_ Ceramics. J. Phys. Chem. C 2018, 122, 15733–15743. 10.1021/acs.jpcc.8b03801.

[ref49] Surta T. W.; Manjón-Sanz A.; Qian E. K.; Mansergh R. H.; Tran T. T.; Fullmer L. B.; Dolgos M. R. Dielectric and Ferroelectric Properties in Highly Substituted Bi_2_Sr(A)TiNb_2_O_12_ (A = Ca^2+^, Sr^2+^, Ba^2+^) Aurivillius Phases. Chem. Mater. 2017, 29, 7774–7784. 10.1021/acs.chemmater.7b02151.

[ref50] Obregón Alfaro S.; Martínez-de la Cruz A.; Torres-Martínez L. M.; Lee S. W. Remove of Marine Plankton by Photocatalysts with Aurivillius-Type Structure. Catal. Commun. 2010, 11, 326–330. 10.1016/J.CATCOM.2009.10.024.

[ref51] Hou D.; Luo W.; Huang Y.; Yu J. C.; Hu X. Synthesis of Porous Bi_4_Ti_3_O_12_ Nanofibers by Electrospinning and Their Enhanced Visible-Light-Driven Photocatalytic Properties. Nanoscale 2013, 5, 2028–2035. 10.1039/C2NR33750A. 23370201

[ref52] Alemi A. A.; Kashfi R.; Shabani B. Preparation and Characterization of Novel Ln (Gd^3+^, Ho^3+^ and Yb^3+^)-Doped Bi_2_MoO_6_ with Aurivillius Layered Structures and Photocatalytic Activities under Visible Light Irradiation. J. Mol. Catal. A: Chem. 2014, 392, 290–298. 10.1016/J.MOLCATA.2014.05.029.

[ref53] Zheng Y.; Duan F.; Chen M.; Xie Y. Synthetic Bi_2_O_2_CO_3_ Nanostructures: Novel Photocatalyst with Controlled Special Surface Exposed. J. Mol. Catal. A: Chem. 2010, 317, 34–40. 10.1016/J.MOLCATA.2009.10.018.

[ref54] Majumdar A.; Pal A. Optimized Synthesis of Bi_4_NbO_8_Cl Perovskite Nanosheets for Enhanced Visible Light Assisted Photocatalytic Degradation of Tetracycline Antibiotics. J. Environ. Chem. Eng. 2020, 8, 103645 10.1016/J.JECE.2019.103645.

[ref55] Lops C.; Ancona A.; Di Cesare K.; Dumontel B.; Garino N.; Canavese G.; Hérnandez S.; Cauda V. Sonophotocatalytic Degradation Mechanisms of Rhodamine B Dye via Radicals Generation by Micro- and Nano-Particles of ZnO. Appl. Catal., B 2019, 243, 629–640. 10.1016/j.apcatb.2018.10.078. 30886458 PMC6420045

[ref56] Yu K.; Yang S.; He H.; Sun C.; Gu C.; Ju Y. Visible Light-Driven Photocatalytic Degradation of Rhodamine B over NaBiO_3_: Pathways and Mechanism. J. Phys. Chem. A 2009, 113, 10024–10032. 10.1021/jp905173e. 19705819

[ref57] Thomas J. M.; Thomas W. J. Principles and Practice of Heterogeneous Catalysis; VCH: Weinheim, 1997, ISBN 3-527-29239-X, Preis: 88, - DM.

[ref58] Saison T.; Chemin N.; Chaneéac C.; Durupthy O.; Ruaux V.; Mariey L.; Maugeé F.; Beaunier P.; Jolivet J. P. Bi_2_O_3_, BiVO_4_, and Bi_2_WO_6_: Impact of Surface Properties on Photocatalytic Activity under Visible Light. J. Phys. Chem. C 2011, 115, 5657–5666. 10.1021/jp109134z.

[ref59] Chen F.; Zhao J.; Hidaka H. Highly Selective Deethylation of Rhodamine B: Adsorption and Photooxidation Pathways of the Dye on the TiO_2_/SiO_2_ Composite Photocatalyst. Int. J. Photoenergy 2003, 5, 209–217. 10.1155/S1110662X03000345.

[ref60] He Z.; Sun C.; Yang S.; Ding Y.; He H.; Wang Z. Photocatalytic Degradation of Rhodamine B by Bi_2_WO_6_ with Electron Accepting Agent under Microwave Irradiation: Mechanism and Pathway. J. Hazard. Mater. 2009, 162, 1477–1486. 10.1016/j.jhazmat.2008.06.047. 18674856

[ref61] Zhang Y.; Zhou J.; Li Z.; Feng Q. Photodegradation Pathway of Rhodamine B with Novel Au Nanorods @ ZnO Microspheres Driven by Visible Light Irradiation. J. Mater. Sci. 2018, 53, 3149–3162. 10.1007/s10853-017-1779-x.

[ref62] Chiu Y. H.; Chang T. F. M.; Chen C. Y.; Sone M.; Hsu Y. J. Mechanistic Insights into Photodegradation of Organic Dyes Using Heterostructure Photocatalysts. Catalysts 2019, 9, 430 10.3390/catal9050430.

[ref63] Lei P.; Chen C.; Yang J.; Ma W.; Zhao J.; Zang L. Degradation of Dye Pollutants by Immobilized Polyoxometalate with H_2_O_2_ under Visible-Light Irradiation. Environ. Sci. Technol. 2005, 39, 8466–8474. 10.1021/es050321g. 16294889

[ref64] Tang R.; Su H.; Duan S.; Sun Y.; Li L.; Zhang X.; Zeng S.; Sun D. Enhanced Visible-Light-Driven Photocatalytic Performances Using Bi_2_WO_6_/ MS (M = Cd, Zn) Heterostructures: Facile Synthesis and Photocatalytic Mechanisms. RSC Adv. 2015, 5, 41949–41960. 10.1039/c5ra04655f.

[ref65] Xue X.; Hanna K.; Deng N. Fenton-like Oxidation of Rhodamine B in the Presence of Two Types of Iron (II, III) Oxide. J. Hazard. Mater. 2009, 166, 407–414. 10.1016/J.JHAZMAT.2008.11.089. 19167810

[ref66] Li D.; Zhou C.; Liang X.; Shi X.; Song Q.; Chen M.; Jiang D. Noble-Metal-Free Mo_2_C Co-Catalsyt Modified Perovskite Oxide Nanosheet Photocatalysts with Enhanced Hydrogen Evolution Performance. Colloids Surf., A 2021, 615, 126252 10.1016/J.COLSURFA.2021.126252.

[ref67] Akbari S. S.; Unal U.; Karadas F. Photocatalytic Water Oxidation with a CoFe Prussian Blue Analogue-Layered Niobate Hybrid Material. ACS Appl. Energy Mater. 2021, 4, 12383–12390. 10.1021/acsaem.1c02188.

[ref68] Maeda K.; Mallouk T. E. Two-Dimensional Metal Oxide Nanosheets as Building Blocks for Artificial Photosynthetic Assemblies. Bull. Chem. Soc. Jpn. 2019, 92, 38–54. 10.1246/bcsj.20180258.

[ref69] Zhou Y.; Wen T.; Zhang X.; Chang B.; Kong W.; Guo Y.; Yang B.; Wang Y. A Multiple Structure-Design Strategy towards Ultrathin Niobate Perovskite Nanosheets with Thickness-Dependent Photocatalytic Hydrogen-Evolution Performance. Chem. - Asian J. 2017, 12, 2727–2733. 10.1002/asia.201701001. 28834347

[ref70] Wang X.; Liu Z.; Liu Z. A Dumbbell CaBi_2_O_4_ Photoelectrode for Photoelectrochemical Water Splitting. ChemCatChem 2017, 9, 4029–4034. 10.1002/cctc.201700938.

[ref71] Kaur A.; Kansal S. K. Bi_2_WO_6_ Nanocuboids: An Efficient Visible Light Active Photocatalyst for the Degradation of Levofloxacin Drug in Aqueous Phase. Chem. Eng. J. 2016, 302, 194–203. 10.1016/j.cej.2016.05.010.

[ref72] He R.; Cao S.; Zhou P.; Yu J. Recent Advances in Visible Light Bi-Based Photocatalysts. Chin. J. Catal. 2014, 35, 989–1007. 10.1016/s1872-2067(14)60075-9.

