# Supplementary material for: Enhanced Photocatalytic Properties of Restacked Unilamellar [SrTa2O7]2– Nanosheets of Aurivillius Phase Layered Perovskites
Source: ACS Omega. 2023 Mar 9;8(11):10607–17. doi: 10.1021/acsomega.3c00593 (PMC10034980; doi:10.1021/acsomega.3c00593)
Supplement: Supplementary file 1 — ao3c00593_si_001.pdf [file ao3c00593_si_001.pdf]

# Enhanced Photocatalytic Properties of Restacked Unilamellar $[SrTa_2O_7]^{2-}$ Nanosheets of an Aurivillius Phase Layered Perovskites

Mohammadreza Khodabakhsh[1], Bengisu Yilmaz[1], Sadegh Firoozi [2], Davoud Fatmehsari Haghshenas[2], Ugur Unal*[1,3]

[1] Chemistry Department, Koc University, Rumelifeneri yolu, Sariyer 34450 Istanbul, Turkey

[2] Department of Materials and Metallurgical Engineering, Amirkabir University of Technology, No. 350, Hafez Ave, Valiasr Square, Tehran, Iran

[3] Koc University Surface Science and Technology Center (KUYTAM), Koc University, Rumelifeneri yolu, Sariyer 34450 Istanbul, Turkey

**Table S1 -** Summary of the composition analysis obtained from XRF of the synthesized powder and restacked nanosheets.

| Sample | Bi (%) | Sr (%) | Ta (%) |
|---|---|---|---|
| As-synthesized Powder | 46.62 | 9.42 | 42 |
| Standard Deviation Error | 0.56% | 0.73% | 0.76% |
| Restacked Nanosheets | 3.64 | 13 | 79.63 |
| Standard Deviation Error | 0.41% | 0.50% | 0.60% |

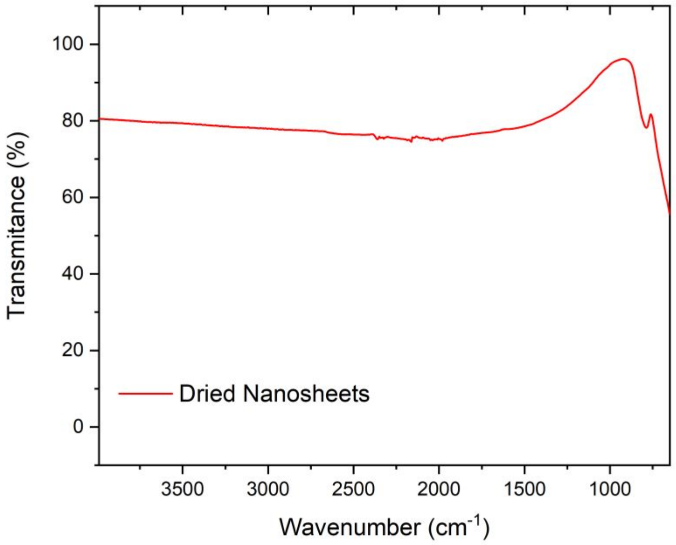


**Figure S1**. FTIR spectrum of the restacked nanosheets after washing with DI water.

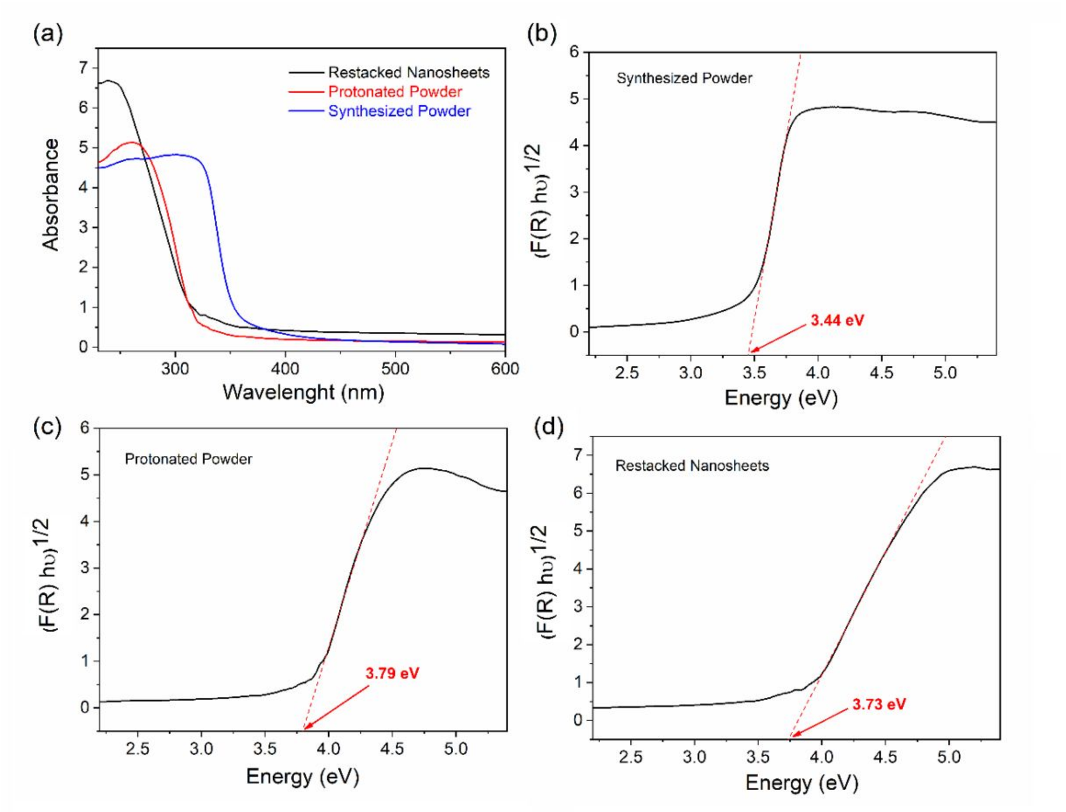


**Figure S2**. Optical band gap measurements. UV−visible spectra (a) and corresponding Tauc plots (b-d) of synthesized powder, protonated powder and restacked nanosheets.

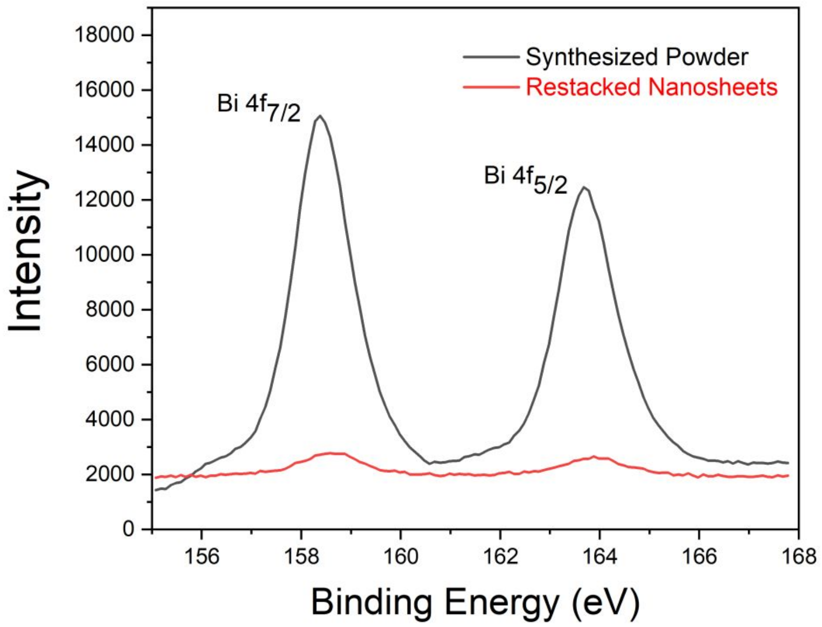


**Figure S3.** Comparison of Bi 4f peaks for the synthesized powder and the restacked nanosheets obtained after exfoliation step confirms the presence of Bi ion in the nanosheets.

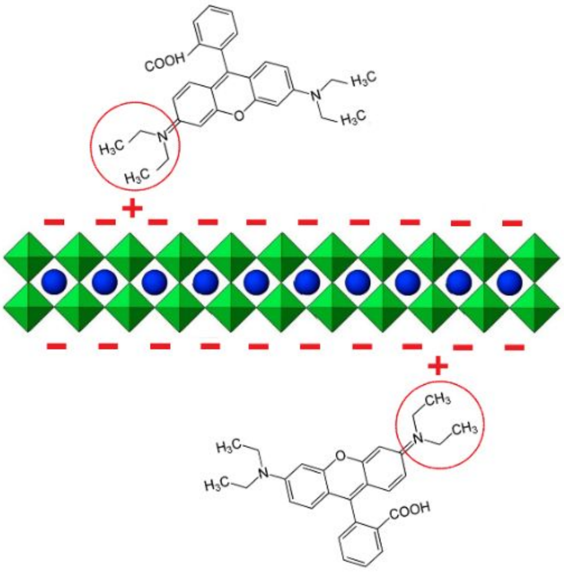


**Figure S4**. Schematic representation of the proposed adsorption mode of Rhodamine B on the surface of the negatively charged nanosheets.

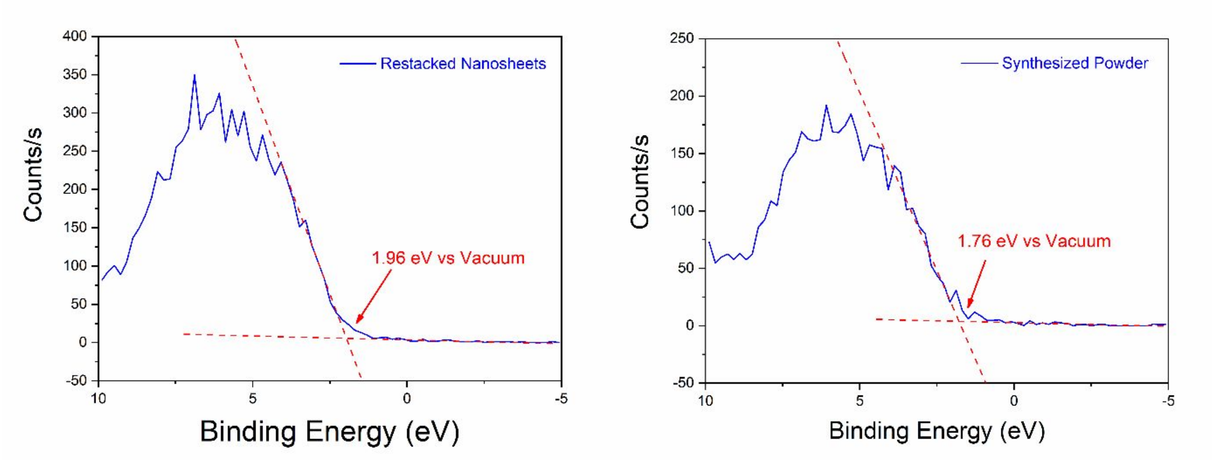


**Figure S5.** Measured XPS valence band spectra for restacked nanosheets (left) and synthesized powder with $[Bi_2O_2]^{2+}$ slabs in interlayers (right).
